# Duodenal Perforation with an Unusual Presentation: A Case Report

**DOI:** 10.1155/2011/512607

**Published:** 2011-09-07

**Authors:** Arif Hussain Sarmast, Fazl Q. Parray, Hakim Irfan Showkat, Yasir A. Lone, Naseer A. Bhat

**Affiliations:** Department of Surgery, Sher-i-Kashmir Institute of Medical Sciences, Soura, Srinagar 190011, Jammu and Kashmir, India

## Abstract

A young female presented with classical complaints suggestive of peptic ulcer disease leading to signs of peritonitis. The said patient after being subjected to baseline workup was subjected to laparotomy which proved to be a surgical surprise. A live ascaris lumbricoides worm was seen pouting out of a duodenal perforation.

## 1. Introduction

Ascariasis continues to be a widespread infection, particularly in Africa, Latin America, India, and Far East. It is estimated that almost 25% of world population are infected with ascariasis. Ascariasis may cause some surgical problems including perforation of duodenal ulcer or intestine [1]. In our valley of Kashmir, ascariasis is endemic especially in pediatric age group. It affects children mainly from low socioeconomic group whose standard of living and hygiene are poor. Poverty unhygienic conditions, sanitation, and unsafe drinking water supply contribute to the spread of infection. The wet soil of Kashmir and temperate climate are excellent condition for the development of larval stage of worms. The adult round worm normally resides in the small intestine but due to their unpredictable dance may even become biliary or pancreatic demons leading to lot many pathologies. Although generally asymptomatic, heavy infestations may cause serious complications like intestinal obstruction, cholangitis, liver abscess, peritonitis, pancreatitis, cholecystitis, and Loffler^,^s pneumonitis [2].

## 2. Case History

A 35 yr old married female presented to our emergency department with complaints of epigastric pain and nausea since 4 hours. She was a known case of peptic ulcer disease and has been erratically on oral Proton pump inhibitors from last 3 years.

A complete workup of patient was done in emergency that revealed pulse rate of 110/minute, blood pressure (BP) = 110/80 mm of Hg, temperature = 99.4°F with mild tenderness and guarding in upper abdomen. Patient was mildly dehydrated. Rest of the systemic examination was normal.

Investigations revealed a hemoglobin (Hb) = 11.4 g/dl, total leukocyte count (TLC) = 8300 cubic mm with polymorphs of 80%, erythrocyte sedimentation rate (ESR) = 35 mm after first hour. Chest X ray (CXR)-standing per abdominal (PA) view and X-RAY abdomen standing and spine views were normal. Ultrasonography (USG) abdomen and pelvis revealed nothing significant. Patient was admitted for further evaluation.

**Figure 1:**
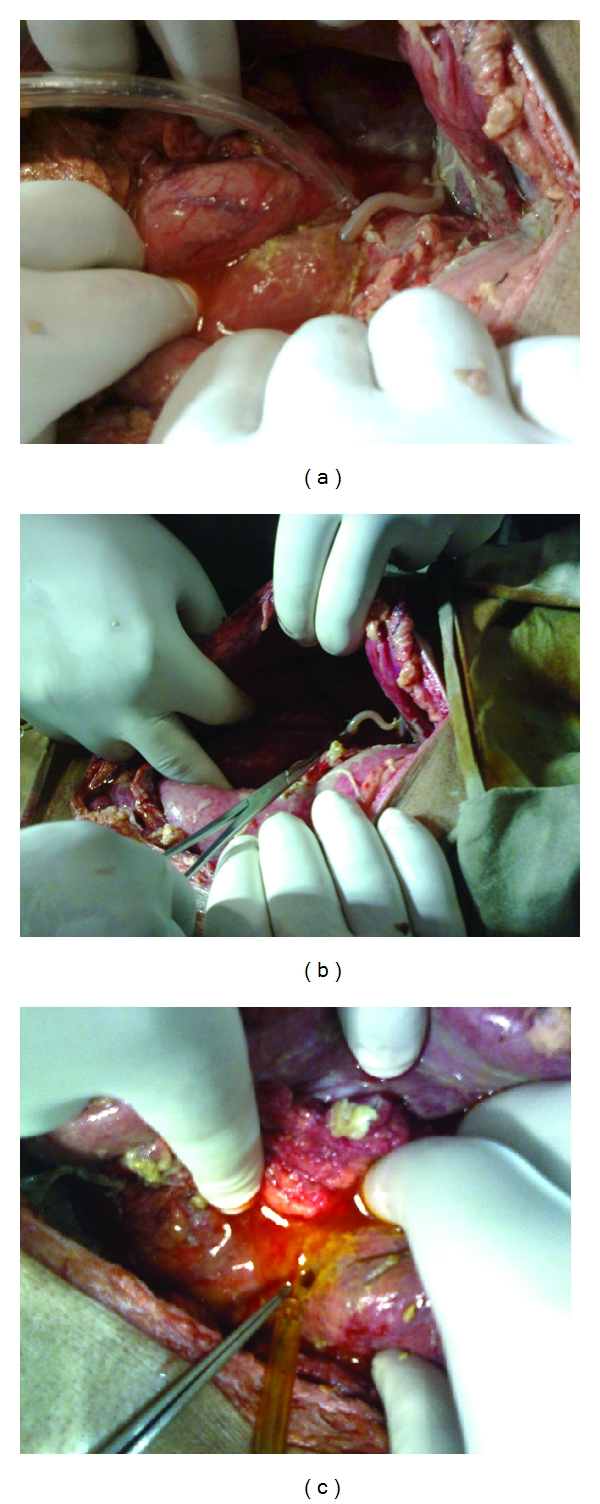


 On 2nd day of admission, patient deteriorated with Pulse rate = 140/min, BP = 100/60 mm of Hg with TLC = 12000 cubic mm, Urea = 58 mg/dL, creatinine = 1.5 mg/dL, and USG revealed free fluid in abdomen. Patient was shifted for emergency laparotomy with a diagnosis of peritonitis possibly due to perforated peptic ulcer. On opening the abdomen, 450 mL of pus was drained, a perforation of about 8 mm diameter was located on the anterior wall of first part of duodenum with a live worm pouting through it (Figure 1(a)).

The worm was manually extracted (Figure 1(b)) after which bile came through the perforation (Figure 1(c)) which was closed with an omental patch with Celin Jone's technique.

The peritoneal cavity was washed thoroughly with a lot of warm saline. The abdomen was closed back with drains in place. Patient was shifted to postoperative ward for further management. The patient's condition improved, and she was discharged home on day 8 after surgery. Whether a preexisting perforation paved the way for the pouting worm or the vice versa cannot be said with certainty. However, there are evidences in literature to suggest that an ascariasis can cause perforation.

## 3. Discussion

Ascariasis is a helminthic infection of global distribution with more than 1.4 billion persons infected throughout the world. The majority of infections occur in the developing countries of Asia and Latin America. Of 4 million people infected in the United States, a large percentage is immigrants from developing countries. Ascaris-related clinical disease is restricted to subjects with heavy worm load, and an estimated 1.2 to 2 million such cases with 20,000 deaths occur in endemic areas per year. More often, recurring moderate infections cause stunting of linear growth, reduced cognitive function, and contribute to existing malnutrition in children in endemic areas. Ascaris infection is acquired by the ingestion of the embryonated eggs. Larvae, while passing through the pulmonary migration phase for maturation, cause ascaris pneumonia. Intestinal ascaris is usually detected as an incidental finding. Ascaris-induced intestinal obstruction is a frequent complication in children with heavy worm load. It can be complicated by intussusceptions, perforation, and gangrene of the bowel [3]. Ascariasis should be investigated in patients with nonspecific abdominal pain or intestinal perforation especially in temperate and tropical countries [1]. The intestine has an immense capacity for dilatation. It has been claimed that it can accommodate >5000 worms without any symptoms [4]. It is thus unlikely that direct pressure by a few *Ascaris *can produce duodenal perforation. The commonest complication of ascariasis is intestinal obstruction due to a worm bolus [5]. The obstruction may be acute or subacute. Acute upper airway obstruction due to roundworms has also been reported [6]. Acute appendicitis and appendicular perforation can occur as a result of worms entering the appendix. Hepatopancreatic ascariasis is a frequent cause of biliary and pancreatic disease in endemic areas [3]. It occurs in adult women and can cause biliary colic, acute cholecystitis, acute cholangitis, acute pancreatitis, hepatic duct calculi, and hepatic abscess [3]. *Granulomatous peritonitis *in ascariasis is not only due to the presence of dead adult worms in the peritoneal cavity [7] but may also be caused by reaction to the eggs [8]. In ascariasis, the cause of perforation of the small intestine remains controversial, with two main theories. In the tropics, patients consistently have histories of diseases associated with the ulceration of the intestines such as typhoid enteritis, tuberculosis, and amebiasis. During extreme conditions, such as inflammation, starvation, or worm bolus obstruction, some parasites are believed to migrate into the ulcers and to cause perforations [7]. Another possible explanation is that the large worm bolus can lead to pressure necrosis and gangrene [9]. The bowel, diseased in this way, becomes susceptible to rupture by the burrowing action of the worm [3, 10].

## 4. Conclusions

Ascariasis as a possible, even though a rare cause should always be borne in mind as a cause of perforation in patients with nonspecific abdominal pain especially in temperate and tropical countries.

## References

[1] Altinel O, Ersoz N, Ozerhan IH (2009). A case of duodenal perforation accompanied by ascariasis. *Journal of Gastroenterology and Hepatology*.

[2] Wani MY, Chechak BA, Reshi F (2006). Our experience of biliary ascariasis in children. *Journal of Indian Association of Pediatric Surgeons*.

[3] Khuroo MS (1996). Ascariasis. *Gastroenterology Clinics of North America*.

[4] Louw M (1966). Abdominal complications of Ascaris lumbricoides manifestation in children. *British Journal of Surgery*.

[5] Basavaraju SV, Hote PJ (2003). Acute GI and surgical complications of Ascaris lumbricoides infection. *Infections in Medicine*.

[6] Lapid O, Krieger Y, Bernstein T, Sofer S, Rsenberg L (1999). Airway obstruction by Ascaris, roundworm in a burned child. *Burns*.

[7] Efem SE (1987). Ascaris lumbricoides and intestinal perforation. *British Journal of Surgery*.

[8] Kinde-Gazard D, Gangbo F, Anagonou S, Gninafon M, Massougbodji A (2000). Granulomatous peritonitis from ascariasis: apropos of 1 case in a Benin child. *Bulletin de la Societe de Pathologie Exotique*.

[9] Surendran N, Paulose MO (1988). Intestinal complications of round worms in children. *Journal of Pediatric Surgery*.

[10] Shoff WH, Steela RW Pediatric association clinical presentation. http://emedicine.medscape.com/article/996482-clinical.

